# Supplementary winter feeding is associated with higher recruitment rates in a population of a scavenging bird of prey

**DOI:** 10.1007/s00442-025-05815-z

**Published:** 2025-10-31

**Authors:** C. Nebel, I. Penttinen, T. Laaksonen

**Affiliations:** 1 Department of Biology, University of Turku, Turku, Finland; 2 Turku Collegium for Science, Medicine and Technology, University of Turku, Turku, Finland

**Keywords:** Conservation management strategies, Settlement, Sex-specific foraging, Population dynamics, Anthropogenic stressors, Scavenging raptors

## Abstract

Supplementary feeding is a widely used conservation strategy to support scavenging species, but the full effects remain poorly understood. In the Baltic Sea region, the recovery of the white-tailed eagle (*Haliaeetus albicilla*) was assisted by an extensive feeding scheme. We examined whether the individual usage of a feeder was associated with the probability to recruit into the local population. At a feeder in Southwest Finland, food was offered during winter, and visiting individuals were identified based on their unique ring codes. In addition, we identified individuals by genotypes extracted from feathers collected from ringed nestlings and from adult feathers collected from nests between 2003 and 2012 and 2008 and 2023, respectively. By matching nestling and adult genotypes, we infer recruitment and relate it to feeder usage. In addition, we explore sex-specific differences, which could arise due to intra-specific competition due to size differences. Our findings reveal that there was high individual variability in feeder usage and that visitation rate was positively associated with the probability to recruit. Although females spent more time at the feeder, recruitment was irrespective of sex. While for individuals the probability to recruit increased from 13.6% to 43.0%, the population-level increases were small, from 13.6% to 14.7%. We demonstrate that winter feeding had the potential to aid the recovery of the white-tailed eagle in the region, although estimating the full effect of supplementary food for the population would require further analyses. Our study highlights the consequences of supplementary feeding and facilitates more informed management strategies for the conservation of endangered species.

**Supplementary Information:**

The online version contains supplementary material available at 10.1007/s00442-025-05815-z.

## Introduction

Globally, wildlife populations are in decline (WWF 2022; IUCN 2024). Conservation interventions are important to halt declines and maintain stable populations, but it is often not clear how effective and well targeted they really are (Le Saout et al. 2013; Larrosa et al. 2016; Rachel et al. 2021).

Supplementary feeding is widely used to support wildlife, from garden birds to game and endangered species (Ewen et al. 2015; Cortés‐Avizanda et al. 2016; Brink et al. 2020). It can improve fitness (Gonzalez et al. 2006; Robb et al. 2008; Fenn et al. 2020), particularly in low-quality natural areas (Rooney et al. 2015; Fenn et al. 2020), but also carries risks, such as disease transmission, exposure to contaminants, or altered ecological interactions and behaviors (Robb et al. 2008; Selva et al. 2014; Plummer et al. 2015; Lawson et al. 2018; Brink et al. 2020). Given these potential benefits and risks, it is important to understand how supplementary feeding influences key demographic parameters such as survival.

Supplementary feeding programs often target scavenging or predatory raptor species to alleviate impacts of anthropogenic pressures (McClure et al. 2018). This species group exhibits delayed maturation, meaning that several years pass before they settle and breed, providing the opportunity to explore age-specific benefits of supplementary feeding. At feeding sites, which often attract many individuals of different species, dominance usually follows intra-specific age and inter-specific body size gradients with the oldest individuals and largest species outcompeting younger individuals and smaller species (Stalmaster and Gessaman 1984; Halley and Gjershaug 1998; Kolodziejczyk et al. 2005; Cortés-Avizanda et al. 2012; Moreno-Opo et al. 2020; but see Gjershaug et al. 2019). In addition, sexual dimorphism is pronounced in raptors (Smith 1999), which raises the potential for competition at feeding sites, with the larger females disadvantaging the smaller males. This could mean that females, due to their larger size and competitive advantage, spend more time at feeding sites, either because they require more food to maintain body condition or because they are able to monopolize access to the resources. It is unclear whether supplementary feeding can reduce these sex-specific differences in access to food resources among threatened species.

The white-tailed eagle (*Haliaeetus albicilla*) is a large sea eagle that inhabits coastal and lakeside environments in the Palearctic, with females being up to 15% larger and 25% heavier than males (Christie and Ferguson-Lees 2010). It predominantly feeds on fish, but will also prey on mammals and birds (Ekblad et al. 2016; Nebel et al. 2023) and does not shy away from scavenging (Christie and Ferguson-Lees 2010). The white-tailed eagle is a conservation success story in Finland, where it almost went extinct in the 1970s (Lokki et al. 2024). Measures were put in place to aid the recovery of the species by the World Wildlife Fund (WWF) Finland: 1) a ringing and monitoring scheme was established that allowed a better assessment of the population, 2) nest sites were protected, and 3) education of the public to increase awareness (Saurola et al. 2003). As the most extensive conservation measure with regard to the amount of work, 4) a winter feeding scheme was initiated in the 1970s, with the aim to provide clean food to white-tailed eagles, and thereby to increase their winter survival. Starting extensively in the 1990s, this also provides a monitoring tool as winter feeding sites allowed ring reading from hides. During the entire project time, there were multiple feeders across Finland, some of them only active sporadically, while at others, food was provided consistently across many years. The white-tailed eagle population recovered and today, the population is no longer considered to be endangered (Högmander et al. 2020), and numbers over 700 known pairs in 2024 (Lokki et al. 2024). The species inhabits coastal areas around the Baltic Sea, sparsely inland habitats in southern Finland, and lakeside environments in Lapland (Lokki et al. 2024). However, there are novel and re-occurring threats such as the rapid development of wind energy (Nebel et al. 2024) and continued exposure to various environmental contaminants (Isomursu et al. 2018; Ekblad et al. 2021; Sonne et al. 2020). The WWF Finland terminated its contribution to the winter feeding already in 2000, but it continued at some sites executed by independent conservationists until March 2013. Winter feeding is believed to have played a major role in saving the Finnish white-tailed eagle from extinction (Stjernberg et al. 2005) by increasing survival rates (Saurola et al. 2003), but this claim has never been formally tested. The Baltic Sea white-tailed eagle population primarily inhabits coastal habitats of the Baltic Sea, but in recent years has started to move further inland to occupy lakeside environments. This dispersal is slow considering the large movement capabilities of the species (Lokki et al. 2024; Penttinen et al. 2024).

In this study, we (1) use resightings from a major winter feeding site in Finland to describe feeder usage across age and sex. In particular, we are interested in sex-specific differences. Given the considerable sexual dimorphism in white-tailed eagles, we predict that larger females spend more time at the feeder than the smaller males. Next, (2) we assess how sex, age, and individual presence at the feeder relate to recruitment into the Finnish population, to test the following hypotheses and predictions: (a) if winter feeding positively influences recruitment, we predict that higher feeder usage will be associated with increased recruitment into the breeding population. Lastly, we look at sex-specific differences: (b) if females outcompete males at the feeder due to the species’ pronounced size dimorphism, this could result in sex-specific recruitment rates that correspond to feeder usage. Our retrospective study will provide insights into the effectiveness of supplementary feeding for scavenging birds of prey, enhancing our understanding of predator and scavenger conservation.

## Materials and methods

### Study area, supplementary feeding and the Yläne feeding site

**Fig. 1:**
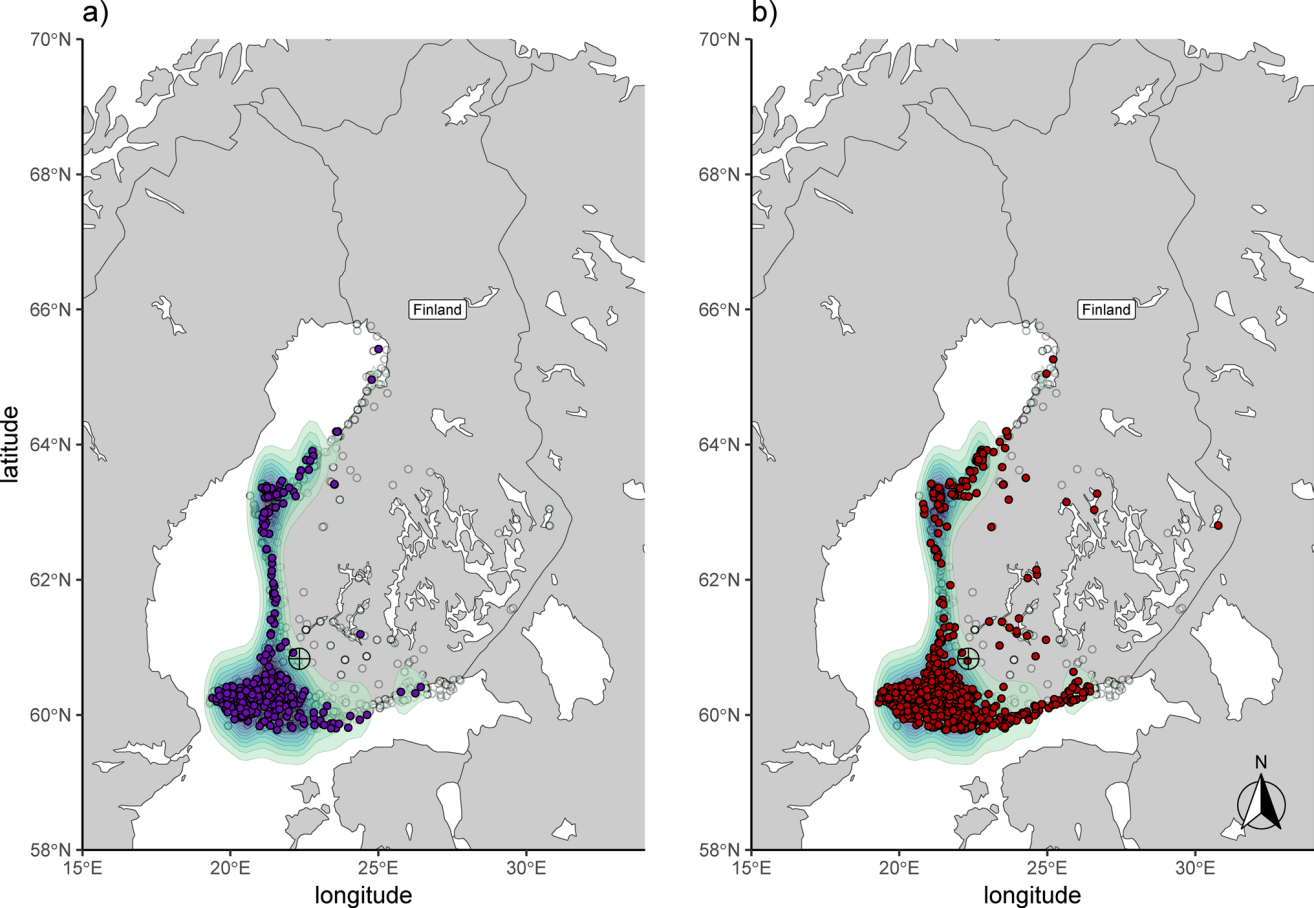
Map showing nest locations of white-tailed eagles that (**a**) produced 1298 nestlings between spring 2003 and 2012 and for which sex and genotype information was available (purple dots) and (**b**) 1110 nests from which adult feathers were collected to infer recruitment (red dots). In both maps, the distribution of nests reflects the distribution of the white-tailed eagle population along the Baltic Sea coast, with a concentration along the coast and in archipelagos and few nests inland (shown by density plot, light colors indicating low density, dark colors high density; light green dots represent the location of all known individual nests). Not shown are nest locations from the northern Lapland area, where another population occurs, but which is genetically distinct from the Baltic Sea population (Ponnikas et al. 2013). The target sign shows the location of the feeding site that operated between the winter 1995/96 and 2012/13 and which was used to assess visitation rate between 2003/04 and 2012/13

Winters in Southwest Finland are characterized by cold temperatures (averages just below freezing but can go as low as -30°C) and snowfall in most years. Lakes and coastal areas are frozen, preventing white-tailed eagles from catching fish, and typical birds used as prey migrate to southern latitudes. Likely, white-tailed eagles rely on natural carcasses and offal left by hunters during that time. In Finland, the first winter feeding was already conducted in the late 1960s, and feeding effort was increased during the 1970s and 1980s, but without systematic ring reading efforts. The Finnish Winter Eagle Project was then formally initiated in the fall of 1990. Across Finland, there were multiple feeding sites that were in use sporadically or during only a few winters. In Southwest Finland, there was one site that was in operation for a prolonged period from 1995 to 2000 (as part of the Winter Eagle Project) and 2001 to 2013 (carried out by independent conservationists): this site was located at 60.8°N, 22.3°E, which is approximately 40 km north of Turku in Yläne (Fig. 1a). There, white-tailed eagles were fed and visiting eagles were identified by reading ring numbers with scopes from hides. Identifications were reported to the Finnish ringing office. Every year, this feeding station was in operation from mid- or end of October until the end of March or mid-April, depending on the severity of the winter.

### Field data collection and data preparation

The monitoring of the Finnish white-tailed eagle population in the Baltic Sea archipelago has been conducted by the White-tailed Eagle working group that has operated under WWF Finland (1973 – 2019) and under the Osprey foundation (2020 onwards). This is an extensive monitoring effort with high coverage of the entire Finnish population. All known territories were visited by volunteers annually during the breeding season (see distribution in Fig. 1a, b), and the majority of known nestlings have been ringed by licensed volunteers in May – early July since 1973 (Stjernberg et al. 2005). The ringing scheme has included one basic metal ring with a unique code on one leg and a color ring with an alphanumeric unique color-engraving combination on the other. To supplement visual resightings with genotype information, volunteers have collected 2 – 3 feathers from the majority of ringed nestlings since 2003 (Fig. 1a) and adult feathers from the nest or beneath it since 2001 (Fig. 1b). In total, 2736 feathers of 1110 nests were collected between 2008 and 2023. Females are more likely to be detected using this methodology, with 59.0% of all detected adults being female (Nebel et al. 2024). A full genotyping and laboratory protocol can be found in the supplementary material. Resighting records of subadult eagles were collected by reading the coded rings at the feeder, whereas resighting records of adult eagles were done visually at nest sites during nest monitoring (from photographs), but primarily through genotyping adult feathers and matching them to the nestling genotypes between 2003 and 2023 (Penttinen et al. 2024). This allowed us to assess recruitment into the Finnish population.

We used the cohorts of white-tailed eagles ringed as nestlings between spring 2003 and 2012 in Finland for the statistical analysis. For these nestlings, we have genotype information, which is used to determine sex and to match nestling and adult samples (Ponnikas et al. 2013; Penttinen et al. 2024). These nestlings also hatched during the active years of the winter feeder that ended in March 2013. The youngest eagles to potentially benefit from feeding (hatched in spring 2012 and using the winter feeder in the winter 2012/13) were 11 years old in the summer of 2023 and therefore old enough to have recruited into the local population. White-tailed eagles reach maturity in their fifth calendar year (Christie and Ferguson-Lees 2010), but in our population, recruitment might only occur when individuals are 8 years or older (unpublished data). Resighting data were obtained from the Finnish Ringing Office. We used resightings that occurred at the Yläne feeder location between October and April, and we removed all individuals (*n* = 13) that were found dead before their first winter.

### Statistical analysis

First, we were interested in whether males and females of different ages exhibited any differences in visitation rates. The response variable was the proportion of human observation days that an eagle was identified (hereafter ‘visitation rate’) in a generalized linear mixed model (GLMM) of the binomial family. The key explanatory variables were sex (factor, ‘male’ or ‘female’), age (continuous, 0 – 9), and their interaction. Age is limited to 0 – 9 years as the observation period covers 10 years, from winter 2003/04 – 2012/13. Furthermore, ringing and observation year were added as random terms to control for different behaviors of cohorts and different winter and feeder conditions, respectively. In addition, we added a random slope for age within individual ID, which allows the effect of age to vary across individuals. A unique observation identifier was added to improve overall model fit.

Second, we were interested in whether recruitment probability was associated with visitation rate, age, and sex. To test this, we fitted a GLMM of the binomial family with recruitment (yes = 1, no = 0) as the response variable. The key explanatory variable was the interactive term sex and the average visitation rate (its calculation is described below) across an individual’s subadult life (age 0 – 4). For this model, we only used the visitation rate of subadults as they have likely not yet recruited. We added the ringing year as a fixed effect (continuous, 2003 – 2012) as younger birds are less likely to have recruited than older birds. As random effects, we added the observation year to account for variation between years and natal territory ID to account for similar behaviors of siblings, e.g., due to the same distance between the natal nest and the feeder.

A potential source of bias arises from birds that were not observed, as those not seen at the feeder could either still be alive but not visiting or could be dead. Since dead birds cannot contribute to recruitment, this introduces the risk of overestimating the results due to this confounding factor. To minimize bias, we implemented the following steps: first, the average visitation rate that was used as a key explanatory variable in the recruitment model was calculated while disregarding years with no observations, ensuring that general survival effects did not skew the results. For example, if a bird had a 0.25 visitation rate in the first year and was not seen again, the average visitation rate would still be 0.25. In addition, we conducted the analysis twice — once including all birds (with results shown in the main text) and once excluding eagles that were never seen at the feeding site (with results shown in the supplementary material). In both cases, we used the same methodology to calculate the average visitation rate (with visitation rate = 0 only assigned to birds that were never seen at the feeder). By repeating the analysis while excluding unobserved birds, we were able to confirm that the results were not influenced by general survival effects, which could lead to the exclusion of dead birds that are unable to recruit.

The GLMM approach with recruitment as a binary response variable was chosen for these data as the majority of resightings occurred at feeders, confounding feeder use and annual survival, and making it difficult to obtain robust survival estimates using traditional mark-recapture models (like Cormack–Jolly–Seber models). However, for this population, where visual identifications of adults are scarce, recruitment based on genotypes is also the best proxy for survival into adulthood.

### Post hoc analyses: population-level effect and settlement distance

To evaluate changes of recruitment due to winter feeding on the population-level (‘average population-level recruitment probability’), we extracted the predicted recruitment rates from the recruitment model. We then used the visitation rates of 1855 individuals that were ringed as nestlings and were observed at the feeder from winter 2003/04 and 2012/13 (including non-observed birds, range = 0 – 0.69, Figure S1a) and the predicted recruitment rates to calculate the population-level average recruitment probability $\overline{R }$ using the following formula:

$$\overline{R }=\frac{1}{N}{\sum }_{i=1}^{N}{R}_{i}$$

where* R*_*i*_ is the predicted recruitment probability for individual *i* from the recruitment model, and *N* is the total number of individuals observed at the feeder.

In addition, we tested whether there were differences in detection probability in relation to distance between the feeder and settlement nest and ringing year by fitting a linear model with distance as the response variable (log-transformed) and mean visitation rate during subadult life and the ringing year as the explanatory variables. Fitting the distance between the feeder and settlement nest allowed us to evaluate whether we were missing any eagles due to emigration to other countries (e.g., Sweden), to where our genotyping effort does not extend. Fitting the ringing year allowed us to assess whether eagles were nesting closer to or further away from the feeder over time, which might be a result of the ongoing population expansion that might result in different detection probabilities and therefore influence the interpretation of our results (Lokki et al. 2024).

All analysis was performed in R v.4.3.0 (R Core Team 2023). For data organization and visualization, we used the ‘tidyverse’ package v.2.0.0 (Wickham et al. 2019); the map was created with ‘sf’ v.1.0-13 (Pebesma 2018), ‘rnaturalearth’ v.0.3.2 (South 2017), and ‘ggspatial’ v.1.1.9 (Dunnington 2023). The distance between the feeder and settlement nest was calculated using ‘geosphere’ v.1.5-18 (Hijmans 2021). The models were fit with the ‘glmmTMB’ package v.1.1.8 (Brooks et al. 2017), and predicted model results were extracted using ‘ggeffects’ v.1.5.0 (Lüdecke 2018); model fit was assessed with the help of the ‘DHARMa’ package v.0.4.6 (Hartig 2022).

## Results

Between spring 2003 and 2012, there were 1855 white-tailed eagles ringed as nestlings in the Baltic Sea area (Fig. 1a). There was considerable variation in the number of resightings among the 1855 individuals, ranging from 1 to 71 days per winter, with a positively skewed distribution (median = 6.0, mean = 8.2 days, SD = 8.1, excluding non-observed birds) (Figure S1a). Of the 1855 ringed nestlings, not all had a feather collected or genotyping failed in the laboratory. Therefore, 1298 had complete information, meaning known genotypes and sex (females = 596, males = 702, with a sex bias towards more males). Of nestlings with complete information, there were 10,641 resightings at the Yläne feeder in the winters between 2003/04 and 2012/13 (at ages 0 – 4, first to fifth winter). There were 774 human observation days in total (mean = 77.4 per winter) (Figure S1b), which is 43% of all 181 days between mid-October and mid-April. The feeder could attract large numbers of ringed eagles during a single day, with an average of 32.4 ringed birds per observation day (ranging from 1 to 109), and the number of individuals identified during one winter averaged 276 (SD = 74) and ranged from 148 (in 2003/04) to 400 (in 2011/12) (Figure S1c, including also birds older than the 2003 – 2012 cohort and eagles ringed between 2003 and 2012 but with no sex or genotype information). It has to be kept in mind that these numbers are an underestimation of true aggregation numbers as we can only account for ringed birds with the data obtained from the Finnish ringing office.

Of 1298 individuals with complete information, 487 (37.5%) were sighted at the feeder between 2003/04 and 2012/13 as subadults (age 0 – 4, first to fifth winter) and 253 (19.5%) recruited (females = 112, males = 104, unidentified sex = 37). Males spent fewer days at the feeder than females (females = 11.2, SD = 11.2; males = 8.63, SD = 9.9, Table 1). There was large individual-level variation in visitation rates, as captured by the random intercept (variance = 1.094, SD = 1.046) and random slope for age (variance = 0.035, SD = 0.188). A strong negative correlation (-0.7) between the intercept and slope indicates that individuals with higher baseline visitation rates experienced steeper declines in visitation rate with age, while those with lower baseline rates exhibited more stable trajectories.

**Table 1:** Results of models explaining winter feeder visitation at a feeding site in Southwest Finland (‘**a**’) and associated recruitment probability (‘**b**’)

	Reference category	ndf	Estimate	SE	χ2	*P*
**a)** **Visitation** **rate,** *N* = 1258						
Sex	Male	**1**	**-0.439**	**0.095**	**21.17**	** <0.001**
Age		**1**	**-0.084**	**0.023**	**13.63**	** <0.001**
Sex * age ǂ		1	**-**0.068	0.039	3.09	0.079
Intercept		**1**	**-2.328**	**0.092**	**640.82**	** <0.001**
**b)** **Recruitment,** *N* = 1298						
Mean visitation rate		**1**	**0.193**	**0.067**	**11.63**	** <0.001**
Sex	Male	1	**-**0.191	0.153	1.565	0.211
Ringing year		**1**	**-0.188**	**0.079**	**5.678**	**0.017**
Visitation rate * sex ǂ	Male	1	**-**0.094	0.120	0.107	0.743
Intercept		**1**	**-1.593**	**0.118**	**181.06**	** <0.001**

ǂnon-significant interactions were removed from the final model

The response variables were (‘**a**’) proportion of human observation days at the feeder that an individual was identified, and the key variable of interested in (‘**a**’) was sex (factor, ‘male’ or ‘female’), age (continuous, 0 – 9) and their interaction. Random effects were observation and ringing year, a random slope for age within individual ID and a unique observation identifier in a binomial GLMM. The response variable for the recruitment model (‘**b**’) was recruitment into the breeder population (binary, 1 or 0). The key variable of interest was the mean visitation rate, and its interaction with sex (factor). The year of hatching was fit as a fixed effect (continuous). Random effects fitted in the model were (‘a’) observation year and individual ID and (‘b’) observation year. Sample sizes are given for each model (*N*), column ‘ndf’ to the degrees of freedom of the numerator. All continuous variables were scaled. Statistically significant explanatory variables are highlighted in bold

**Fig. 2:**
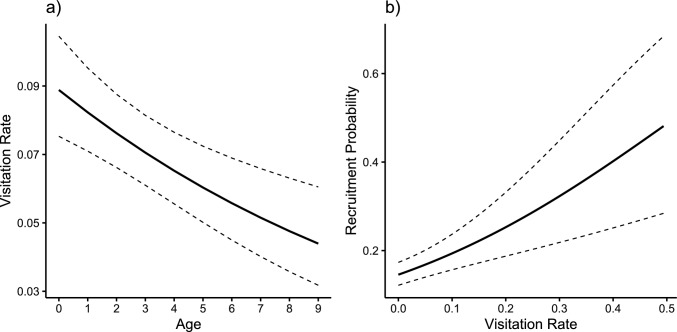
Effect sizes of GLMMs. (**‘a’**) Age-specific (0 – 9 years, first to tenth winter) visitation rate of 1298 eagles ringed as nestlings between 2002 and 2012 and seen in winters 2002/03 to 2012/13; (**‘b’**) their recruitment probability in relation to mean visitation rate during subadult years (0 – 4, first to fifth winter). Visitation rate is the proportion of human observation days that an eagle was identified in one winter, and data used to plot the graph include unobserved birds. Error bars show 95% confidence interval

We found indications that winter feeding was positively associated with the probability to recruit into the Finnish population: eagles that spent more days at the feeder were significantly more likely to be confirmed as breeding birds in Finland (Table 1, Fig. 2b). Effects were significant in the datasets including and excluding unobserved birds (c.f. Table 1, S1). Predicted recruitment probabilities increased from 0.14 (confidence interval [95% CI] = 0.11–0.17; min. visitation rate = 0) to 0.43 (CI = 0.24–0.64; max. visitation rate = 0.53) (Fig. 2b). The interaction of visitation rate and sex was non-significant, meaning that there were no sex-specific benefits of feeder usage on recruitment (Table 1). The average recruitment probability was 0.147 for each individual, which means that winter feeding likely contributed to a population-level recruitment increase from 13.6% to 14.7%, meaning a 1.1% increase. Of recruited individuals, there was no effect of settlement distance and the mean visitation rate (χ2 = 1.53, *P* = 0.216) or ringing year (χ2 = 0.37, *P* = 0.543).

## Discussion

High visitation rates at a supplementary feeding site were associated with a higher probability to recruit into the local population of white-tailed eagles than eagles visiting the feeder less often. As recruitment is a key fitness component in life history, this positive association indicates that birds that fed more from feeders were at an advantage over those that spent less time there. Females and young birds used the feeder more often than males and older birds, respectively, but sex-specific visitation did not translate into sex-specific benefits of feeder usage on recruitment.

### Winter feeding as a successful conservation tool?

Winter feeding was implemented as a protective measure to increase survival when persecution and contaminants had caused a near-extinction of the white-tailed eagles in the Baltic Sea region in the 1970s (Saurola et al. 2003). Our results indicate that feeder usage is associated with an increase in recruitment probability, rising from 15% if birds never used the feeder to 43% if they were present during more than half of all observation days. However, due to the strong positive skewed data distribution of visitation rate and only a few individuals using the feeder on more than a few winter days, this only translates into an increase of 14.6% (no birds using feeders) to 15.7% (birds using feeders according to observed frequencies) on the population level, a 1.1% increase. Although this difference appears minor, even slight demographic changes can have profound impacts on long-lived and slowly reproducing species like the white-tailed eagle (Congdon et al. 1994; Webb et al. 2002; Tack et al. 2017). It also needs to be kept in mind that the observation period (years 2003 – 2012) does not match with the times of greatest distress for the Finnish population and it is possible that beneficial effects were more pronounced in times of the all-time population low in the 1970s – 1980s and during times of rapid population growth in the 1990s and 2010s (Stjernberg et al. 2005; Lokki et al. 2024).

### Recruitment is a combined consequence of survival and site fidelity

Recruitment reflects a combination of demographic and behavioral components, including survival and site fidelity, which means that there is an alternative mechanistic explanation for the observed result: eagles that use winter feeders more frequently might have a higher affinity to Finland and therefore recruit more likely into local territories than emigrate. If this is indeed the case, then the observed higher recruitment might not be the result of higher survival, but lower probabilities to settle in nearby counties. While supplementary feeding has been shown to influence both survival (Oro et al. 2008; Fenn et al. 2020) and movement behaviors (Robb et al. 2008; López-López et al. 2014; Plummer et al. 2015) in various species, including white-tailed eagles (Rajchard and Procházka 2009), our post hoc analysis of detection probability indicates that visitation rate as subadults does not affect settlement distances. Eagles that visited feeders more frequently did not settle closer to the site, suggesting that winter feeding did not alter settlement behavior or reduce the probability for long-distance dispersal and therefore emigration. Nonetheless, supplementary feeding remains a plausible driver of alternative settlement behaviors in white-tailed eagles and other scavenging species due to its broader impact on survival and movement patterns.

### Individual, sex- and age-specific usage of the winter feeder

The Yläne feeder could attract individuals in great numbers each observation day. As we were only able to account for ringed eagle presence with the data obtained from the Finnish ringing office, this is an underestimate of true aggregation numbers, which also include unringed birds. The feeder usage patterns of white-tailed eagles offer intriguing insights into potential behavioral dynamics: there was large individual variability in feeder usage. Females used the feeder consistently more often than males, and the visitation rate in males declined faster than in females with age. Potentially, the observed pattern reflects different winter foraging strategies with females prioritizing secure food sources to ensure their survival, whereas males utilize smaller or scattered resources. Similar strategies have been shown in differently sized vulture species (Moreno-Opo et al. 2020). Winter feeder usage declined with age, with high-visiting individuals experiencing more pronounced declines over time. This decline with age is somewhat counterintuitive to the idea that older birds outcompete younger birds (Stalmaster and Gessaman 1984; Kolodziejczyk et al. 2005) and should therefore monopolize feeding sites. Whether this indicates that young birds need to compensate for competition against older birds by spending more time at the feeder or that older birds are not relying on feeders as much due to better foraging efficiency remains speculative in the absence of behavioral observations of antagonistic interactions and additional movement information.

## Conclusion

White-tailed eagles are numerous in Finland today (Lokki et al. 2024), but as for other large birds of prey, there are novel anthropogenic threats to the population like the development of wind energy (Balotari-Chiebao et al. 2021; Nebel et al. 2024) or bioaccumulating environmental toxins (Ekblad et al. 2021; Sonne et al. 2020). Understanding the main contributors to the population increase is important to ensure effective conservation of the species in the future. We found that the extensive winter feeding scheme implemented as a conservation effort likely had a positive effect on the white-tailed eagle population by increasing the probability of recruitment into the population. At the same time, feeding might also be able to continue the population increase even when it is no longer needed in terms of conserving the population. Supplementary feeding is a relatively common practice to support wild raptor and carnivore populations, which are at increased risk due to anthropogenic activities and climate change (McClure et al. 2018). Understanding the impact of supplementary feeding is therefore crucial for conservation efforts as it can influence population dynamics, distribution patterns, and ultimately species viability. By assessing these effects at the population level, conservationists can implement targeted management strategies to enhance the long-term sustainability of vulnerable wildlife populations.

## Supplementary Information

Below is the link to the electronic supplementary material.

Supplementary file1 (DOCX 137 KB)

## Data Availability

Data are available in Nebel et al. (2025).
