# Supplementary material for: Supplementary winter feeding is associated with higher recruitment rates in a population of a scavenging bird of prey
Source: Oecologia. 2025 Oct 31;207(12):181. doi: 10.1007/s00442-025-05815-z (PMC12578676; doi:10.1007/s00442-025-05815-z)
Supplement: Supplementary file 1 — Supplementary file1 (DOCX 137 KB) [file 442_2025_5815_MOESM1_ESM.docx]

SUPPLEMENTARY MATERIAL

**Supplementary winter feeding is associated with higher recruitment rates in a population of a scavenging bird of prey**

***DNA extraction and genotyping lab procedure***Laboratory work for the collected feather samples was carried out by the Center of Evolutionary Applications (University of Turku, Finland). DNA from adult feather samples was extracted with a method modified from (Aljanabi and Martinez 1997). For microsatellite analysis, we used two multiplex PCRs and amplified one locus separately: individuals were genotyped by using 14 loci (Multiplex 1: Hal-01, Hal-07, Hal-09, Hal-03, Hal-13, Aa-27, IE-12, Hal-04, Hal-05; Multiplex 2: IE-04, Hal-06, Aa-11, IE-11; single Hal-14). Microsatellites were amplified in 10 µl reaction volumes using QIAGEN Multiplex PCR kit (Qiagen Inc. Valencia, CA, USA). Primer concentrations were 0.15 µM for all primers, except for Hal-14, for which a primer concentration of 0.20 µM was used following the standard protocol. To improve the microsatellite peak profiles, a GTTT-tail was added to the 5’ end of each reverse primer (Brownstein et al. 1996). The following PCR profile was used: 94°C for 15 min followed by 34 cycles of 94°C for 30 s, annealing in 58°C for 90 s and 72°C for 60 s and a final extension in 72°C for 10 min. The sex of the samples was determined by amplifying short diagnostic fragments on the Spindlin gene (marker Z37B) in an additional reaction using the same standard protocol with annealing temperature at 58°C (Dawson et al. 2015).

Amplifications were performed on Bio-Rad S1000 and Applied Biosystems 2720 thermal cyclers and the size of the fragments was determined by capillary electrophoresis on an ABI PrismTM 3130xl genetic analysis instrument. The peak profiles of the pooled samples could then be separated during scoring and visual inspection, using GeneMarker version 2.4.0 (SoftGenetics). Initially, all samples were run in duplicates but due to the high consistency between them (of 398 individuals run in duplicates, translating into a combined non-exclusion probability of identity of 6.5 * 10^-9^, (Jamieson and CS Taylor 1997; Waits et al. 2001), samples were run a single time consequently. Genotypes with less than 9 successfully amplified loci were excluded. We screened the genotypes extracted from adult feathers for matches from samples obtained from nestlings. To do so, we used the software Cervus 3.0.7 (Kalinowski et al. 2007) with settings of a minimum of 9 matching loci and one mismatch allowed.


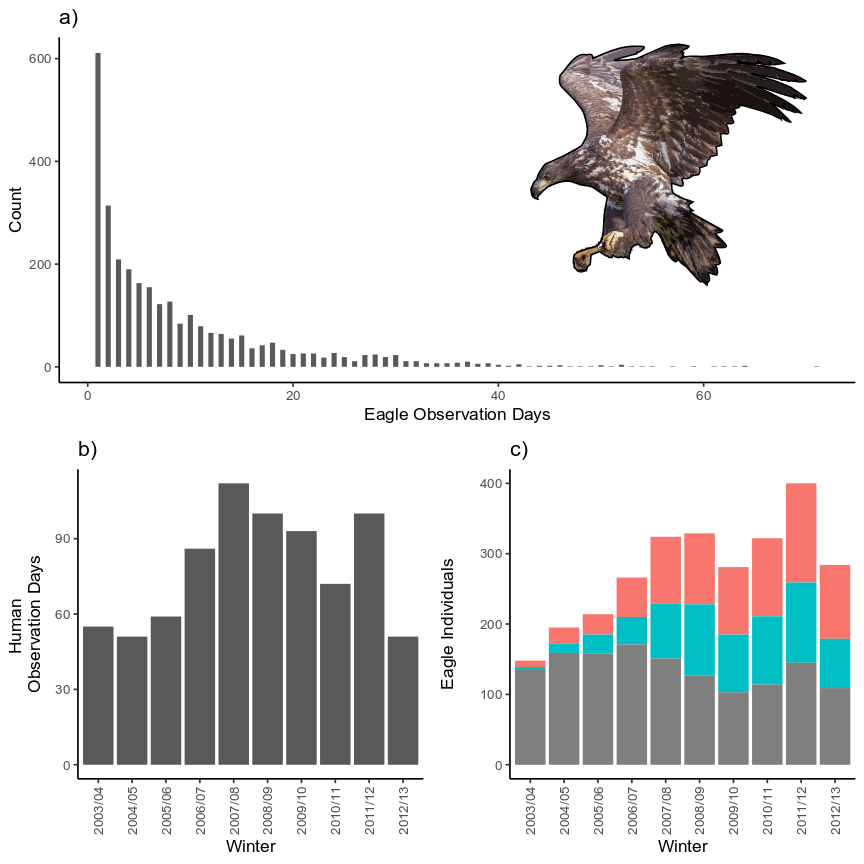


**Figure S1** Description of observation data and visitation behavior of white-tailed eagles observed at the winter feeder between the winter 2003/04 and 2012/13. (**a**) Histogram of the number of observation days across 1855 ringed individuals and ages (excluding unobserved birds), (**b**) number of days eagles were identified by volunteers at the feeder each winter (‘human observation days’), (**c**) females (red color), males (blue) and eagles of unknown sex (grey) identified each winter. The count in (c) includes birds ringed before 2003 – 2012, explaining the high proportion of unknown sex and increase in birds with known sex.

**Table S1** Results of models explaining recruitment by winter feeder visitation at a feeding site in Southwest Finland. The response variable was recruitment into the breeder population (binary, 1 or 0). The key variable of interest was the mean visitation rate, and its interaction with sex (factor). The year of birth was fit as a fixed effect (continuous). Random effects fitted in the model were observation year and natal territory ID. Column ‘ndf’ gives the degrees of freedom of the numerator. All continuous variables were scaled. Statistically significant explanatory variables are highlighted in bold. Excluded are birds that were never observed at the feeder.

|  | Reference  category | ndf | Estimate | SE | χ2 | P |
| --- | --- | --- | --- | --- | --- | --- |
| **Mean visitation rate** |  | **1** | **0.255** | **0.108** | **5.63** | **0.018** |
| Sex | Male | 1 | -0.181 | 0.223 | 0.66 | 0.417 |
| **Ringing year** |  | **1** | **-0.263** | **0.114** | **5.35** | **0.021** |
| Visitation rate * Sex ǂ | Male | 1 | -0.084 | 0.130 | 0.146 | 0.703 |
| **Intercept** |  | **1** | **-1.228** | **0.152** | **65.13** | **< 0.001** |

ǂ non-significant interaction removed

**References**

Aljanabi SM, Martinez I (1997) Universal and rapid salt-extraction of high quality genomic DNA for PCR-based techniques. Nucleic acids research 22:4692–3

Brownstein MJ, Carpten JD, Smith J R (1996) Modulation of non-templated nucleotide addition by Taq DNA polymerase: primer modifications that facilitate genotyping. BioTechniques 20:1004–1010

Dawson DA, Brekke P, Dos Remedios N, Horsburgh GJ (2015) A marker suitable for sex-typing birds from degraded samples. Conservation genetics resources 7:337–343

Jamieson A, CS Taylor S (1997) Comparisons of three probability formulae for parentage exclusion. Animal genetics 28:397–400

Kalinowski ST, Taper ML, Marshall TC (2007) Revising how the computer program CERVUS accommodates genotyping error increases success in paternity assignment. Molecular Ecology 16:1099–1106

Waits LP, Luikart G, Taberlet P (2001) Estimating the probability of identity among genotypes in natural populations: cautions and guidelines. Molecular Ecology 10:249–256
